# Barriers and Facilitators of Nurses’ and Physicians’ Willingness to Work during a Respiratory Disease Outbreak: A Mixed-Methods Systematic Review

**DOI:** 10.3390/ijerph18136841

**Published:** 2021-06-25

**Authors:** Hyun Jie Lee, Eunkyung Kim, Brenna L. Morse, Seung Eun Lee

**Affiliations:** 1 Severance Hospital, 50-1 Yonsei-ro, Seodaemun-gu, Seoul 03722, Korea; uni_chae@yuhs.ac; 2 College of Nursing, Yonsei University, 50-1 Yonsei-ro, Seodaemun-gu, Seoul 03722, Korea; kek1005@yuhs.ac; 3 Solomont School of Nursing, University of Massachusetts Lowell, 113 Wilder Street, Lowell, MA 01854, USA; Brenna_Morse@uml.edu; 4 Mo-Im Kim Nursing Research Institute, College of Nursing, Yonsei University, 50-1 Yonsei-ro, Seodaemun-gu, Seoul 03722, Korea

**Keywords:** nurses, physicians, willingness, respiratory disease outbreak, barrier, facilitator

## Abstract

This review was undertaken to identify the perceived barriers and facilitators of nurses’ and physicians’ willingness to work during a respiratory disease outbreak. This mixed-methods systematic review involved the extraction of data from the electronic databases PubMed, Web of Science, CINAHL, and PsycINFO and from a manual search of articles published between 2003 and April 2021. The quality of the included studies was assessed using a mixed-method appraisal tool. A total of 29 studies were eligible for inclusion: 21 quantitative and 8 qualitative. Using the Integrated Behavioral Model, perceived barriers and facilitators were identified under seven categories: demographics, attitude, perceived norm, personal agency, knowledge and skills to perform the behavior, environmental constraints, and habit. The results of this study broaden the understanding of various factors that affect nurses’ and physicians’ willingness to work during a respiratory disease outbreak. These findings will facilitate the modification of current pandemic workplace staffing strategies and practices and will inform preparedness planning for similar situations in the future.

## 1. Introduction

During a disease outbreak, increased capacity in terms of personnel, equipment, supplies, and structure is needed to address surge capacity across health facilities [1]. Although all resources are important, adequate staffing and availability of healthcare is necessary to address patient and community needs during a respiratory disease outbreak, such as the most recent coronavirus disease (COVID-19) pandemic. It is essential to secure sufficient numbers of healthcare workers, including nurses and physicians, during a respiratory disease outbreak [2], as it is the workers and not the facility beds, ventilators, or other equipment that actually provide frontline care. Nurses and physicians are usually expected to sacrifice their own health and well-being for the benefit of patients during a public health emergency such as the COVID-19 pandemic [3]. Many ethical and societal factors contribute to the notion or the need to sacrifice and can vary between providers and facilities. However, through research focused on the severe acute respiratory syndrome (SARS) outbreak [4], the H1N1 influenza pandemic [5], and the COVID-19 pandemic [6], researchers have demonstrated that nurses or physicians may refuse to participate in caring for patients who are infected or suspected to be infected with novel diseases during outbreaks. For example, in a recent study, approximately 23% of psychiatrists and nurses in China expressed unwillingness to care for patients infected with the severe acute respiratory syndrome coronavirus 2 (SARS-CoV-2), which causes COVID-19 [7]. In an Australian study, about 39% of the nurses reported that they were unwilling to provide care for patients in intensive care units during the COVID-19 pandemic [8].

Willingness refers to “an individual’s openness to opportunity” [9] (p. 896) and is the most influential predictor of individuals performing a specific behavior [10]. The willingness of nurses and physicians to work refers to their intention or wanting to provide care for patients during the pandemic [8]. We acknowledge that facility surge capacity and the unavailability of needed supplies have complicated healthcare professionals’ work experiences, optimism, rates of burnout, and other aspects of pandemic care; however, the healthcare personnel’s willingness to work can affect public access to healthcare and the quality of the care in a disaster [11]. Thus, it is necessary to understand the willingness of healthcare personnel to engage in patient care amid extreme challenges, such as respiratory disease outbreaks, to inform future pandemic planning [12]. To date, there is limited literature on nurses’ and physicians’ willingness to provide patient care during a respiratory disease outbreak.

Researchers have identified several factors associated with healthcare workers’ willingness to work during an influenza pandemic. For example, personal characteristics (e.g., gender and occupation), knowledge and skill-related factors (e.g., clinical knowledge of influenza pandemics, confidence in personal skills, role-specific knowledge, and having pandemic response training), perceived safety factors (e.g., perceived personal safety, awareness of pandemic risk, and knowledge of the peak phase of the influenza emergency), concern for family and loved ones, and personal obligations have affected healthcare workers’ willingness to work during an influenza pandemic [13,14]. Healthcare team members have a duty to provide direct patient care, and therefore are at higher risk of getting infected with a respiratory virus because of the time expended in implementing bedside care [3]. Nurses and physicians provide considerable personal, hands-on patient care. The factors that influence the willingness of nurses and physicians to provide such care may differ from factors that other inter-professional healthcare team members consider important.

There is a critical need to identify barriers and facilitators to nurses’ and physicians’ willingness to work during a disease outbreak. The identification of these factors can help healthcare systems, leaders, stakeholders, and policymakers to address barriers and facilitators in order to support adequate availability of the requisite care during a respiratory disease outbreak [13,15,16]. A literature review identified factors related to healthcare workers’ willingness to work during an influenza pandemic through an analysis of quantitative studies [13]. However, it is valuable to review both qualitative and quantitative studies as it enriches the evidence, which in turn provides broad perspectives and strong rationale for improved decision making [17]. Therefore, we undertook a mixed-method systematic review to provide a rich and highly practical understanding of a complex issue [18]: the willingness of healthcare workers to work during a pandemic. Understanding the factors related to nurses’ and physicians’ willingness to engage in patient care during a respiratory disease outbreak will facilitate the modification of current pandemic workplace practices and may serve to inform future pandemic planning.

## 2. Materials and Methods

We followed a mixed-methods systematic review procedure that was described by Pluye and Hong [18]. In addition, we used the Preferred Reporting Items for Systematic Reviews and Meta-Analyses (PRISMA) checklist [19] and the Enhancing Transparency in Reporting the Synthesis of Qualitative Research Guidance (ENTREQ) [20] to structure reporting and guide the synthesis of study findings.

Prior to conducting the literature search, we searched the International Prospective Register of Systematic Reviews and Cochrane Library for related work on our topic and ascertained that there were no similar systematic reviews. A systematic search of the literature was conducted during April 2021, using the PubMed, Web of Science, CINAHL, and PsycINFO electronic databases. We used specific search strategies because of the differences in the search process across databases. Table S1 lists the search strategies that were undertaken. Key search terms were related to population (i.e., nurse, physician, healthcare worker, healthcare provider), willingness to work (i.e., willingness, willing* and work, report to work, respon* and work), and respiratory disease outbreaks (i.e., pandemic, healthcare disaster, respiratory infectious disease epidemic, and disease outbreak*). The ancestry approach was conducted to identify other articles that met the inclusion criteria and contributed to the aim of this review. The literature searches were conducted in consultation with a medical librarian. 

The inclusion criteria for the present review specified studies that: (1) focused on nurses’ and/or physicians’ willingness to work during a respiratory disease outbreak, (2) were original research published in a peer-reviewed scholarly journal, and (3) were published in English. Studies were excluded if they were theoretical, discussion, or review articles or if they focused on non-respiratory diseases, such as Ebola virus.

**Figure 1:**
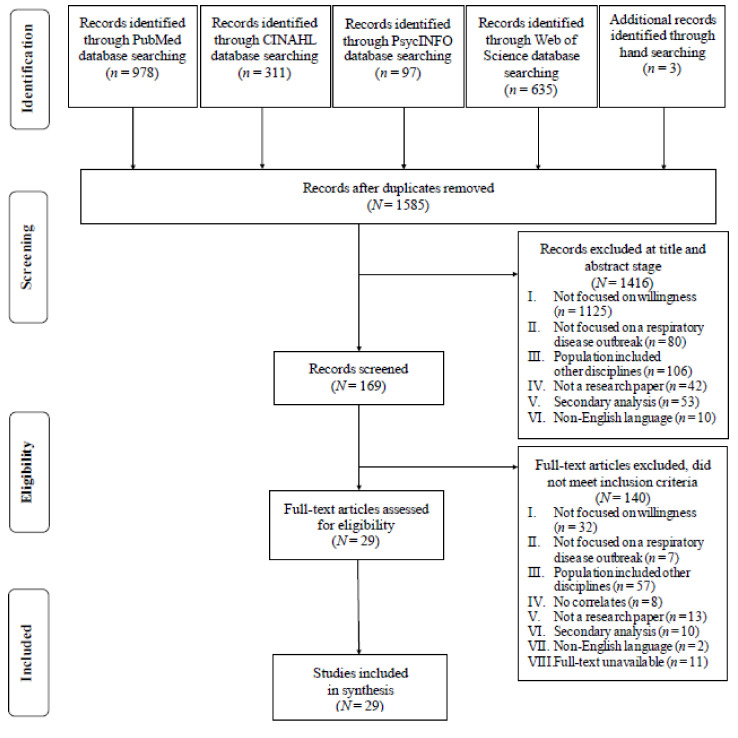
Preferred Reporting Items for Systematic Reviews and Meta-Analyses Flowchart of Article Selection for Analysis.

The PRISMA framework guided the article search and review processes as shown in Figure 1. The initial database search yielded 2021 articles. A manual search identified 3 additional articles. After excluding duplicates, 1585 articles remained. Following title and abstract review by two researchers (HJL and EK) independently, 1416 articles were excluded, as they did not meet the inclusion criteria. A total of 169 articles were included for full-text review, of which 140 articles were excluded. A final sample of 29 studies were included in this review; 8 articles were qualitative studies and 21were quantitative studies.

The quality of the selected articles was appraised using the mixed-method appraisal tool (MMAT), which evaluates the methodological quality of qualitative, quantitative, and mixed-methods studies [21]. Each study was reviewed using MMAT for clarity of the research question and the sufficiency of the collected data to address the research question. Then, according to each study design, the quality of the study was evaluated with five questions that had three possible responses (yes, no, or cannot tell). Using a blinded process, two researchers (HJL and EK) evaluated the quality of each manuscript that was included. When the reviewer ratings were discordant, the reviewers discussed their findings until reaching a consensus on the quality of all articles.

In the data extraction process, one researcher (HJL) extracted data and another researcher (EK) validated the data extraction. Data from the 29 included studies were organized using a matrix of the following characteristics: first author, year of publication, country, type of study, study aim, sample and setting, and main results (Table 1).

Data synthesis was based on a sequential explanatory synthesis [18]. To enable this synthesis, results of quantitative studies were pooled in evidence tables based on identified perceived barriers and facilitators of nurses’ and/or physicians’ willingness to work. Findings of qualitative studies were integrated using a qualitative thematic analysis (Table 2). After themes were identified, the theme was compared with the barriers and facilitators identified during quantitative synthesis.

## 3. Results

### 3.1. Study Characteristics

A total of 29 articles were included in this review: 8 qualitative research articles and 21 quantitative research papers. The included manuscripts were published between 2003 and 2021. As shown in Table 1, studies were conducted in various countries, including Australia (*n* = 4) [8,22,23,24], Bangladesh (*n* = 1) [25], Canada (*n* = 1) [26], China (*n* = 7) [2,7,27,28,29,30,31], Georgia (*n* = 1) [32], Hong Kong (*n* = 1) [33], Nigeria (*n* = 1) [34], Pakistan (*n* = 1) [35], Philippines (*n* = 1) [36], Qatar (*n* = 1) [37], South Korea (*n* = 2) [38,39], Taiwan (*n* = 3) [4,40,41], Turkey (*n* = 1) [42], USA (*n* = 3) [5,43,44], and Yemen (*n* = 1) [15]. Among the 29 studies reviewed, 19 studies included nurses only [4,5,8,22,27,28,29,30,31,33,36,37,38,39,40,41,42,43,44], 6 included physicians only [23,24,25,26,34,35], and 4 included both nurses and physicians [2,7,15,32]. A total of 19 studies were conducted during an outbreak or pandemic, such as the SARS outbreak (*n* = 2) [4,41], the H1N1 influenza pandemic (*n* = 3) [5,33,43], the Middle East respiratory syndrome (MERS) outbreak (*n* = 1) [39], and the COVID-19 pandemic (*n* = 13) [2,7,8,25,27,28,29,30,31,35,36,37,42]. One study was conducted to evaluate the high possibility of an avian influenza (AI) outbreak [40]. The other studies were conducted using hypothetical influenza pandemic scenarios. One study [33] was conducted in a community setting, whereas the others were conducted in the hospital setting. MMAT ratings were higher than 80% for most of the studies reviewed. No study was excluded based on the quality rating. Table S2 displays the MMAT ratings for all included studies.

### 3.2. Summary of Evidence

**Figure 2:**
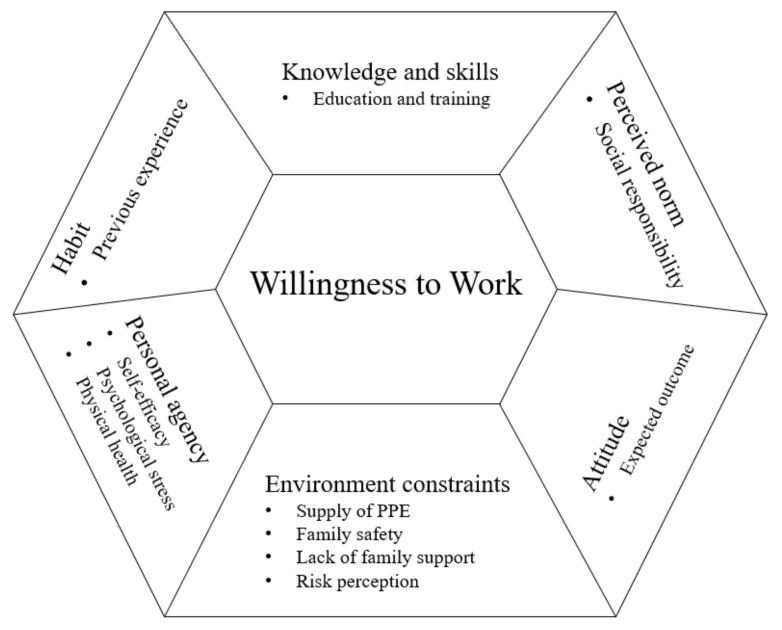
Barriers and Facilitators of Nurses and Physicians’ Willingness to Work During a Respiratory Disease Outbreak.

We integrated the findings of the reviewed studies using the Integrated Behavioral Model (IBM), which is a theoretical construct that presenting how individual motivational factors affect the willingness to perform a specific behavior [10]. In this review, we summarized the perceived barriers and facilitators of nurses’ and/or physicians’ willingness to work in seven categories adopted from the IBM: demographics, attitude, perceived norm, personal agency, knowledge and skills to perform the behavior, environmental constraints, and habit (Figure 2). The attitude represented instrumental attitude, which is determined by beliefs about the outcomes of the behavior. Perceived norms include social identity, which is the social pressure one feels to perform or not perform a particular behavior. In this review, social responsibility and professionalism, which includes putting the patient’s interests ahead of their own [38], are perceived as societal norms among healthcare workers. Personal agency refers to an individual’s capability to perform a behavior for given purposes, which comprises self-efficacy and perceived control over behavioral performance. Knowledge and skills to perform the behavior included respiratory disease outbreak-response training. Environmental constraints refer to physical constraints that hinder a behavior. In this review, we divided environmental constraints into physical constraints and emotional constraints in a respiratory disease outbreak situation. Lastly, when the behavior is habitual and the person has previously performed such behavior, that particular behavior is more likely to occur [10]. This review replaced habit with the past experience of participants.

#### 3.2.1. Demographics

Gender, age, professional experience, education level, occupation, and participating in religious activities influenced nurses’ and physicians’ willingness to work during a respiratory disease outbreak. Three research teams concluded that, compared with women, men were more likely to be willing to work if an influenza pandemic occurred [25,26,34]. Research teams evaluated findings related to chronological age. The younger that the physicians were, the more willing they were to work during the COVID-19 pandemic [25]. Similarly, nurses who were older than 40 years were less willing to work compared with nurses aged 20–29 years [28]. In terms of the duration of professional experience, nurses who had worked for 11–15 years were less willing to participate in frontline pandemic work than nurses with 21 or more years of experience [31]. The findings of another research study supported the finding that senior nurses were more likely to report willingness to combat COVID-19 at the professional frontline [28]. Nurses with higher levels of education were more willing to work during the pandemic [29]. When analyzed by occupation, nurses were less willing to work than physicians in case of a hypothetical influenza pandemic [32]. Among physicians-in-training and more senior physicians, those who were no longer in training reported greater unwillingness to work during the COVID-19 pandemic [25]. In addition, regardless of the religion practiced, nurses who engaged in religious activities were more likely to be willing to work during a possible avian flu pandemic [40].

#### 3.2.2. Attitude

Nurses’ and physicians’ willingness to work was affected by the expected outcomes of providing care during a respiratory disease outbreak, such as infection, overwork (e.g., workload inequities, care assignments with mismatched needs and resources available, or exploitation), quarantine, and incentives. Researchers determined that nurses hesitated to participate in caring for patients suffering from MERS due to fear of infection [39]. During the H1N1 influenza pandemic, community nurses who were more fearful of infection reported greater unwillingness to work [33]. Similarly, general practitioners were hesitant to care for patients during a possible pandemic as they prioritized family safety and did not want to carry a virus to their families [23]. The nurses who thought COVID-19 affected their workload had a lower level of willingness to work [29]. Moreover, nurses’ willingness to work significantly decreased when there was a need to quarantine during and after providing care for patients infected with respiratory viruses [4,5,41].

A research team that studied the impact of incentives on willingness concluded that when double wages were provided, when accommodation was provided, and when vaccines and medicines were provided, not only to nurses themselves but also to their families, the willingness of nurses to work decreased [43]. In contrast, the nurse participant of one study stated that she would not participate in the next outbreak because she was not adequately compensated after taking care of patients with MERS [39]. General practitioners who were not currently in clinical practice stated that professional risk and liability played an important role in their willingness to provide care during a possible influenza pandemic [24].

#### 3.2.3. Perceived Norm

Eleven studies identified social responsibility as a facilitator of nurses’ and physicians’ willingness to work [2,23,24,27,29,30,36,38,39,42,44]. Nurses with higher scores of social responsibility were more likely to report their willingness to work if a respiratory outbreak occurred [38,44]. Nurses and physicians have been reported to have a strong sense of responsibility to care for patients infected during a pandemic [2,39]. Furthermore, the physicians believed that refusal to work during a pandemic was an abandonment of their responsibilities to both their patients and colleagues [23,24]. Some nurses learned about the difficulties that healthcare workers and patients were experiencing during the COVID-19 pandemic from news broadcasts and social media, which was antecedent to their decision to volunteer to support the COVID-19 care sites to extend help [27].

#### 3.2.4. Personal Agency

Nurses and physicians with high self-efficacy were more likely to work during an influenza pandemic [15]. Those who were confident in their knowledge of the risks of COVID-19 and how to protect themselves and their patients from infection reported that they would care for COVID-19 patients [7,25,31,37].

Stress and other psychological issues that nurses experienced during a respiratory disease outbreak reduced the nurses’ willingness to work [28,29,33,38]. Multiple research teams have determined an inverse relationship between high levels of stress among nurses and willingness to work during an outbreak [33,38]. The nurses who felt anxious and depressed during the COVID-19 outbreak reported a lower level of intention to respond to calls for work during subsequent waves of COVID-19 [28,29]. Moreover, the perceived physical health of nurses influenced their willingness to work. Nurses who felt energetic and spirited or in stable conditions of health were more likely to volunteer to work in the COVID-19 medical sites [28].

#### 3.2.5. Knowledge and Skills to Perform the Behavior

Training to improve knowledge and skills that nurses and physicians can use to address patient needs with a disease outbreak was associated with their willingness to work. For example, after providing a 4 h online and a 4-day field training about the influenza pandemic for nurses, the confidence and willingness of nurses to work during a pandemic significantly increased [22]. Psychiatrists and nurses who completed a training program had a greater likelihood of accepting an assignment that included infected patients [7]. Having protective equipment training had a positive effect on nurses’ intention to respond during the COVID-19 pandemic [28,29]. Nurses in intensive care units (ICUs) who perceived that they received enough information from the organization regarding SARS-CoV-2 transmission, restrictions due to the risks of COVID-19, use of personal protective equipment (PPE), availability of relevant education, and access to mental health services were more willing to provide nursing care during the pandemic [8]. The ICU nurses’ intention to work was unassociated with the actual preparedness of the ICU to manage COVID-19 surges [8]. Nurses who perceived that they were well-prepared against emergency situations such as the COVID-19 pandemic reported a high level of intention to work [29]. When physicians noted inadequate training regarding care of COVID-19 patients, they were more reluctant to treat patients with the illness [35].

#### 3.2.6. Environmental Constraints

The availability of adequate PPE (e.g., masks, disposable gowns, and gloves) was identified as an important factor in willingness to work. For instance, inadequate supply of PPE prevented nurses and physicians from being willing to work during respiratory disease outbreaks [5,23,24,35,40,43]. General practitioners stated that they would stop working in an expected pandemic if PPE was unavailable [24]. However, some general practitioners stated that they would work regardless of whether PPE was provided adequately [24]. When nurses agreed with infection control principles, they were more likely to report to work during an outbreak [4,41]. Similarly, physicians who believed that using PPE would keep healthcare workers safe from getting COVID-19 tended to have greater willingness to work [25].

Nurses having family at risk for illness or death were less likely to work in an influenza pandemic [5,43]. Emergency nurses with children in the home [44] and female physicians who needed to care for family [26] were significantly less likely to report to work during an expected outbreak. Furthermore, physicians who had an elderly relative at home expressed their reluctance to treat COVID-19 patients [35]. Similarly, worries about family care and lack of family support were barriers affecting nurses’ willingness to work during the COVID-19 pandemic [31]. Nurses reported increased willingness to volunteer to work in the COVID-19 pandemic when their families had a supportive attitude toward working in the COVID-19 medical sites [28].

Fear about an outbreak was negatively associated with nurses’ willingness to work during a threatened outbreak [40], and increased worry among nurses about an outbreak was related to a decreased likelihood of reporting to work [44]. Furthermore, frightening news reports about a pandemic as well as a hypothetical situation where a 30-year-old healthcare colleague died from workplace disease exposure reduced nurses’ willingness to work [5,43]. In contrast, low self-perceived risk of a SARS-CoV-2 infection in the workplace was a predictor of nurses’ and physicians’ willingness to work during the COVID-19 pandemic [25,31].

#### 3.2.7. Habit

The lived experience of caring for patients who were infected or suspected of being infected with the pathogen implicated in an outbreak positively affected the willingness of nurses and physicians to work. Five studies determined that prior experience in caring for patients infected or suspected of being infected during a respiratory disease outbreak was positively associated with willingness to work in the same or a similar future outbreak [7,15,29,38,39]. Past experiences increased the confidence of working during an outbreak, which in turn increased the willingness to work [39]. However, one research team determined that physicians who treated confirmed or suspected COVID-19 patients had less willingness to continue their work during the COVID-19 pandemic, whereas physicians who had the experience of treating patients during previous pandemics (e.g., H5N1 or H1N1) had greater willingness to work with COVID-19 patients [25].

## 4. Discussion

This mixed-methods systematic review identified barriers and facilitators that influence the willingness of nurses and physicians to work during a respiratory disease outbreak. Key barriers included attitude toward expected negative outcomes resulting from working during the outbreak; environmental constraints, including inadequate supply of PPE; concern of family; and perceived risk. Key facilitators were perceived norms (e.g., social responsibility or duty as nurses and physicians); personal agency, including self-efficacy, knowledge, and skills improved through training; and habits influenced by previous experiences.

The perceived norm, a duty as nurses and physicians, was an important role in their willingness to sacrifice themselves in a respiratory disease outbreak. However, their duty was grounded in a reciprocity for their acceptance of greater risk for the public good [45]. For example, nurses and physicians who provide direct care to infected patients should be prioritized in the allocation of scarce medical resources such as PPE during the COVID-19 pandemic [46]. In the same line with reciprocity, the environmental constraints were major barriers of willingness to work in a respiratory disease outbreak. In addition, nurses’ and physicians’ attitudes toward the negative expected outcomes of providing care during a respiratory disease outbreak, including infection and quarantine, decreased their willingness to work. In order to facilitate nurses’ and physicians’ willingness to work in an outbreak, it is essential to support their safety and their ability to protect themselves from infection. For a safe workplace, provision of adequate PPE is essential. The British Medical Association, Canadian Nurses Association Code of Ethics, and Royal College of Nursing highlight the reciprocal duty of employers and governments to protect nurses and physicians by providing necessary and sufficient protective equipment and supplies during disasters, outbreaks, and pandemics to minimize the healthcare workers’ risk of infection [47,48,49]. Recently during the COVID-19 pandemic, there was a global lack of adequate PPE for frontline healthcare workers [50]. In March 2020 (in the early days of the COVID-19 pandemic), there was a massive personnel recruitment in the National Health System in the UK due to concerns that physicians would quit their jobs due to fear of inadequate supplies of PPE [51]. The supply and management of adequate PPE is a task for the government and not one for individual facilities or workers because the antecedent of most PPE shortages during the COVID-19 pandemic was mainly due to insufficient stockpiles and limited manufacturing capacity [52]. For example, after a SARS outbreak, the government of Taiwan developed a stockpiling system of PPE that could maintain a minimum stockpile for addressing the surge in demand for PPE in the early stages of a pandemic [53], and this strategy was helpful in coping with the COVID-19 pandemic [54]. The South Korean government took several steps to actively resolve the shortage of PPE in healthcare settings and the community. The government purchased 80% of the masks from domestic manufacturers, banned exports of PPE, and limited the price of masks and the amount of masks that an individual could buy every week [55]. India, which relied on imports for the supply of PPE, started producing and manufacturing PPE with various government institutes and some private manufacturers, given the poor quality of imported PPE [56]. National agencies are encouraged to check and improve the government stockpiling and manufacturing system with regard to PPE based on the lessons learned from the ongoing COVID-19 pandemic. A proactive approach will support worker safety and reduce the situational constraints that hinder willingness to work.

In addition, this review identified that training to cope with an outbreak facilitated willingness to work during a respiratory disease outbreak. Providing nurses and physicians not only with adequate PPE but also with sufficient information and resources may help alleviate fears related to working during a disease outbreak. Furthermore, the training increased the confidence of nurses and physicians to work in the COVID-19 pandemic situation, which positively affected their willingness to work [7]. In this review, confidence and self-efficacy as a personal agency facilitated their willingness to work. In a study in Australia, field and online training increased nurses’ willingness to work in the outbreak, as did knowledge about how to deal with an outbreak situation [22]. According to a review on implementing disaster and pandemic training programs for medical students, a program including 1-day training improved disaster and pandemic preparedness, attitude toward working in disaster and pandemic situations, and knowledge and skills [57]. In addition, a 1 h computer-based simulation training enhanced nurses’ self-efficacy and working skill in a disaster [58]. Simulation-based training is increasingly used in disaster management not only to educate caregivers with the required knowledge and skills but also to give them experience in handling a disaster situation, which is one of facilitators of nurses’ and physicians’ willingness to work [58]. In pandemics, there can be a lack of training about the specific infection and PPE best practices for healthcare workers, and some trainings may not be mandatory [59]. We suggest that organizations provide an outbreak-specific simulation training for nurses and physicians to improve their outbreak situation-related knowledge, skills, and confidence, which will support their willingness to work. Such trainings provide important information and serve as a reliable source of knowledge during a time when myths and rumors may be prevalent, and therefore participation in such trainings should be obligatory.

Most of the studies we reviewed had several limitations. First, the studies including both nurses and physicians did not explore the differences among the two groups of professionals. Occupation or role-related barriers and facilitators of willingness to work during a pandemic should be determined to develop an effective workforce strategy. Second, most of the studies that were analyzed in this review used quantitative methodology. However, willingness is an individual perception that is influenced by various factors [9]; therefore, it may be best to explore this concept with mixed-methods or fully qualitative work. The qualitative studies enriched the evidence that professional responsibility facilitated a willingness to work. Finally, there is insufficient evidence to report with confidence that researchers have obtained a comprehensive understanding of the various barriers and facilitators of willingness to work in a pandemic situation. For example, in this review, the results of qualitative and quantitative studies on the impact of rewards were inconsistent and limited, which makes it difficult to describe the influence of a reward on the willingness to work during an outbreak. To determine best practices for compensation and support for working during a pandemic, we suggest the need for additional research on how and what type of compensation affects the willingness to work in a respiratory infectious disease outbreak. Such research should be approached from the perspective that fair work deserves fair pay and that the increase in stress, workload, and responsibility during a pandemic undoubtedly warrants additional compensation.

There are several limitations of this review. As the literature search was limited to records published in English, we may have missed important and relevant studies published in other languages. This review included studies with low MMAT quality scores. However, the results were derived by synthesizing and analyzing results across 29 studies, most of which had quality ratings of 80% or higher. Although this review included studies conducted during an actual respiratory disease outbreak situation and those conducted with hypothetical prompts, we did not analyze situation-related influencing factors. Additionally, there are different healthcare systems in each country according to their socio-economic situation. For example, government healthcare support and spending, as well as individual or family funds available to support health needs, vary between OECD and non-OECD nations; the funding models are one source of disparity that impacts health indicators such as life expectancy and infant mortality [60]. The nature of the different healthcare systems may affect nurses’ and physicians’ willingness to work. However, this review did not consider differences among the healthcare systems. Therefore, in order to develop more specific interventions, we suggest that future studies consider health system differences between countries along with the factors that influence willingness to work, whether such studies are conducted in the context of an actual outbreak situation or a hypothetical situation. Nevertheless, this review broadens the understanding of perceived facilitators and barriers for willingness to work among nurses and physicians in a respiratory disease outbreak. 

## 5. Conclusions

This review provides an understanding of the barriers and facilitators affecting nurses’ and physicians’ willingness to work during a respiratory disease outbreak. Individual workers across different health systems and settings will have variability in the resources afforded them to carry out healthcare, and they will approach care with differing professional lived experiences. Regardless of the evidence-based policies and programming on the willingness to work that are studied and implemented, it is important to respect and respond to nurses’ and physicians’ concerns about their safety and preparedness when providing care during a respiratory disease outbreak. The environmental constraints that could harm the health of nurses and physicians and concerns regarding family wellbeing had a negative impact on the willingness to work. Factors that have a positive effect on the willingness to work include a sense of social responsibility and self-efficacy in terms of adequate knowledge and skills needed to provide care for patients during an outbreak. Establishing a working environment that is safe from infection by facilitating the consistent availability of proper PPE is a prerequisite for supporting worker well-being and willingness to work. Even in usual non-pandemic situations, education and training for nurses and physicians should be regularly implemented in preparation for an unexpected respiratory disease outbreak.

## Figures and Tables

**Table 1:** Summary of Studies Reviewed.

No.	First Author (Year)	Study Type	Aims	Sample/Setting	Main Results
1	Adam [34] (2014)	Quantitative descriptive	To assess the knowledge level of physicians, their preparedness to respond to an influenza pandemic, and the preventive practices employed	Physician (*n* = 240) Hospital (*n* = 1) Nigeria using hypothetical situation	A total of 60.4% of physicians had fair knowledge about influenza. Only 10% had a positive attitude about working during an influenza pandemic. Female physicians were less willing to report to work in the event of a pandemic than male counterparts (*p* = 0.001). Officers and registrars (physicians who have not yet completed training) were less willing to report to work than consultants (physicians who have completed training and registration) (*p* = 0.001).
2	Al-Hunaish [15] (2019)	Quantitative descriptive	To assess healthcare workers’ willingness to participate in biological and natural disasters and to identify associated factors	Nurse (*n* = 381) Physician (*n* = 311) Hospital (*n* = 3) Yemen using hypothetical situation	A total of 90% of the participants expressed high willingness to participate in any type of disasters, 77.3% against natural disasters, and 66% against an influenza pandemic. High trust in work safety was positively associated with willingness to participate in any type of disaster (OR ^2^ = 2.535, *p* = 0.004). Males were more willing to participate in a natural disaster (OR ^2^ = 1.639, *p* = 0.015). Previous experience working through a pandemic was positively associated with willingness to participate in an influenza pandemic (OR ^2^ = 1.528, *p* = 0.024). Self-efficacy was associated with willingness to participate in a disaster response for any type of disaster (OR ^2^ = 1.319, *p* < 0.001), natural disasters (OR ^2^ = 1.143, *p* < 0.001), and an influenza pandemic (OR ^2^ = 1.114, *p* < 0.001).
3	Anikeeva [23] (2008)	Qualitative	To explore general practitioners’ perceptions of their preparedness for an influenza pandemic, the changes they would make to their practice, and the ethical justifications for their planned actions	General practitioner (*n* = 10) Hospital (*n* = NS ^1^) Australia using hypothetical situation	Themes identified: Preparedness for a pandemic: no major changes in their practice, difficult to manage the impact on their practice Changes to the operation of general practices: importance of minimizing physical contact with influenza patients, as this is the most effective way of reducing disease transmission Personal protective equipment (PPE): the government has a reciprocal duty to ensure that working conditions are safe Antiviral medication: families having access to the medication is an important determining factor to work during an influenza pandemic General practitioners’ justification of planned actions: general sense of commitment to the public good, moral obligation vs. responsibility to oneself to stay healthy and to protect their own family.
4	Ayub [35] (2020)	Quantitative descriptive	To explore the concerns of physicians in the context of the COVID-19 pandemic and to evaluate the reasons for their reluctance to treat patients	Physician (*n* = 208) Hospital (*n* = 7) Pakistan during COVID-19 pandemic	A total of 83.7% of the respondents expressed reluctance to treat patients with COVID-19. Having elderly relatives at home (*p =* 0.001), no prior training to deal with COVID-19 patients (*p =* 0.016), and unavailability of masks and PPE (*p =* 0.010) were associated with their reluctance.
5	Bell [44] (2014)	Quantitative descriptive	To assess the perceived likelihood of emergency nurses reporting to work during an avian influenza (AI) outbreak and to explore the Protection Motivation Theory (PMT) constructs as predictors of reporting to work	Nurse (*n* = 353) Hospital (*n* = NS^1^) USA using hypothetical situation	A negative relationship was identified between the willingness to work and worry about an AI outbreak (*r* = −0.12, *p* = 0.039). Nurses who lived with children were less likely to report to work (β = −0.14, *p* = 0.01). Social responsibility was significantly related to willingness to work (*r* = 0.18, *p* = 0.01). The information sources associated with reporting to work included formal training while on the job (β = 0.12, *p* = 0.03) and membership in a professional organization (β = 0.14, *p* = 0.01). Five of the PMT constructs combined accounted for 37% of the variance in willingness to work.
6	Butsashvili [32] (2007)	Quantitative descriptive	To determine the factors associated with likely absenteeism of hospital-based healthcare workers during a potential influenza pandemic	Nurse (*n* = 158) Physician (*n* = 130) Hospital (*n* = 2) Georgia using hypothetical situation	Females indicated they were less to working during the pandemic than male respondents (RR ^3^ = 2.95, 95% CI ^4^:1.13–7.7), and nurses were less willing than physicians (RR ^3^ = 2.04, 95% CI ^4^: 1.26–3.29).
7	Cui [27] (2020)	Qualitative	To explore the experiences and psychological adjustments of nurses who voluntarily traveled to Hubei Province in China to provide support during the COVID-19 epidemic	Nurses (*n* = 12) Hospital (*n* = NS ^1^) China during COVID-19 pandemic	Themes identified: Motivations for supporting the hardest-hit areas included professional commitment, family support, media propaganda Challenges faced during the support missions were heavy workloads, changes in working patterns, communication barriers, barriers associated with wearing PPE Psychological experiences such as uncertainty, fear of infection, loneliness, stressful events, sleep disorders Psychological adjustments, including decreased anxiety following adequate training and PPE availability, professional instinct triumphing over fear, positive responses to stress, and social support Personal and professional growth through a stronger professional identity, positive work attitude, harmonious interpersonal relationship, expended possibilities, living and learning, cherishing life
8	Dickinson [26](2013)	Quantitative descriptive	To investigate family physicians’ willingness to work during an influenza pandemic	Physician (*n* = 192) Hospital (*n* = NS ^1^) Canada using hypothetical situation	More than half the physicians (78% of males, 60% of females) responded they would be willing to continue working during an influenza pandemic. Males were more willing to continue working than females. In some situations, physicians who trained in South Africa and Britain and physicians who worked in rural sites were more willing to continue working than other groups.
9	Gan [28] (2020)	Quantitative descriptive	To investigate the willingness of Chinese nurses to practice in Hubei combating the coronavirus disease 2019 and to explore the associated factors	Nurse (*n* = 11183) Hospital (*n* = NS ^1^) China during COVID-19 pandemic	A total of 83.4% of the nurses were willing to volunteer to practice in Hubei during the COVID-19 pandemic. Location, age, professional qualification, working department, political party membership, marital status, attitude of families, training, time spent on learning related knowledge, health condition, and anxiety levels were associated with willingness to volunteer to practice in Hubei (all *p* < 0.05).
10	Hope [22] (2011)	Quantitative non randomized	To determine the appropriateness of engaging advanced nurses as public health surge staff and to determine whether a training changed perceptions and confidence toward working during an influenza pandemic	Nurse (*n* = 54) (clinical nurse consultants, nurse educators, and nurse managers) Hospital (*n* = NS ^1^) Australia using hypothetical situation	After an educational intervention, self-perceived knowledge and confidence in providing nursing care during an influenza pandemic and willingness to respond to future pandemic increased (*p* < 0.01).
11	Kim [39] (2018)	Qualitative	To identify psychological stress in nurses who cared for MERS-CoV (MERS) patients and to identify systemic problems of the Korean healthcare system nurses experienced during pandemic work	Nurse (*n* = 12) Hospital (*n* = 4) South Korea during the MERS outbreak	Themes identified: 1. Going into a dangerous field - New challenge: nurses were tired of their original departments - Hesitancy and hoping to avoid: nurses did not want to work due to fear of infection, but they were compelled to go - Strong responsibility as a nurse: nurses considered pandemic work to be unavoidable and inevitable given their professional responsibilities 2. Strong pressure because of MERS-CoV - Inevitable fear due to lack of information and frequently-changing guidance on infection control protocols - Being alone in isolation rooms - Exhausted strength and extreme stress: nurses became exhausted from the patient care and felt more stress - Stigma from society, even in a hospital 3. The strength that makes me endure - Comradery with fellow healthcare workers - The patient whom I have to care for: nurses felt pity for the patients - CEncouragement: nurses experienced a changed view of society from negative perceptions to positive ones 4. Growth as a nurse - Constant mind control - Nursing: Lighting up the dark; nurses were proud of the meaning of nursing and their job as nurses 5. Remaining task - Futility of forgotten warriors: nurses felt that rewards were not adequate for their efforts during pandemic work - Building a preparation system - Expectation about changed perception: people’s attitudes needed to be changed to follow the instructions of medical providers
12	Li [29] (2020)	Quantitative descriptive	To assess the current level of emergency preparedness and to identify associated factors of intention to respond and emergency preparedness of nurses during COVID- 19 pandemic	Nurse (*n* = 1646) Hospital (*n* = NS ^1^) China during COVID-19 pandemic	Moral consideration, the level of emergency preparedness (EP), being treated differently by society, previous participation in COVID- 19 protection training, working experience in SARS, overwork, education level, intention to leave, support of a public nurse, feelings of anxiety and depression, and working department explained 34.6% of the total variance in intention to respond (IR) model (*F* = 80.05, *p* < 0.001). EP significantly predicted IR (β = 0.20, *p* = 0.001). Pathway analysis revealed that moral consideration, intention to leave and impacts on work and life mediate the relationship between EP and IR.
13	Liu [2] (2020)	Qualitative	To describe the experiences of healthcare providers in the early stages of the COVID-19 outbreak	Nurse (*n* = 9) Physician (*n* = 4) Hospital (*n* = 2) China during COVID-19 pandemic	Themes identified: 1. Being fully responsible for patients’ wellbeing: “this is my duty” - A call to duty: joining the fight - Treating and caring for patients: managing both mundane and extraordinarily difficult situations - Supporting patients emotionally: “treating the patient, not just the disease” 2. Challenges of working on COVID-19 wards - Working in a completely new context - Overwhelmed and exhausted by the workload and protective gear - The uncertainty and fear of being infected and infecting others - Witnessing patients’ experiences (in a good way and bad way) - Relationship between patients and healthcare providers: trying to engage amid chaos 3. Resilience amid challenges - Many sources of social support to cope with the situation (social, organizational level, colleague, individual level) - Transcendence
14	Liu [30] (2020)	Qualitative	To explore the experiences of front-line nurses combating the COVID-19 epidemic	Nurse (*n* = 15) Hospital (*n* = 2) China during COVID-19 pandemic	Themes identified: 1. Facing tremendous new challenges and danger - New challenge - Hoping to avoid infection 2. Strong pressure because of COVID-19 - Inevitable fear - Exhaustion - Extreme stress 3. Strong responsibility and identity as a healthcare provider - Responsibility and mission as a healthcare provider - Nursing: Lighting up the dark 4. Rational understanding of the epidemic - Hopeful - Expectation about disaster rescue training - Improving rescue preparation system
15	Lord [8] (2021)	Quantitative descriptive	To assess intensive care unit (ICU) nurses ’ willingness to provide nursing care for a patient with COVID-19 during the first few weeks of the COVID-19 pandemic in Australia	Nurse (*n* = 83) Hospital (*n* = 1) Australia during COVID-19 pandemic	A total of 61% of the nurses were willing to provide nursing care for a patient in the ICU. There were positive correlations between willingness to provide nursing care and knowledge of the COVID-19 pandemic (*r* = 0.388, *p* < 0.001) and communication from managers (*r* = 0.399, *p* < 0.001). There was a negative correlation between willingness to provide nursing care and personal concerns (*r* = −0.271, *p* = 0.013). There was no association between willingness to provide nursing care and nurses perception of the preparedness of the ICU (*r* = −0.135, *p* = 0.223). Communication from managers was the only predictor of willingness to provide nursing care (β = 0.172, *p* = 0.031).
16	Luo [31] (2021)	Quantitative descriptive	To explore the current status of Chinese nurses’ willingness to work during the COVID-19 pandemic and the factors that influence them	Nurse (*n* = 1310) Hospital (*n* = 6) China during COVID-19 pandemic	A total of 90.5% of nurses reported that they were willing to work on the front-line of the pandemic. The factors affecting nurses’ willingness to work were 11–15 years of experience (OR ^2^ = 0.313; 95% CI ^4^: 0.160–0.609), having previous infection prevention training (OR ^2^ = 0.472; 95% CI ^4^: 0.29–0.766), self-efficacy (OR ^2^ = 1.130; 95% CI ^4^: 1.058–1.207), perceived risk (OR ^2^ = 0.813; 95% CI ^4^: 0.711–0.929), perceived self-worth (OR ^2^ = 1.903; 95% CI ^4^: 1.477–2.451), worries about family care (OR ^2^ = 0.672; 95% CI ^4^: 0.520–0.870), and worries about lack of family support (OR ^2^ = 0.714; 95% CI ^4^: 0.559–0.913).
17	Martin [5] (2011)	Quantitative descriptive	To determine factors affecting nurses’ ability and willingness to work during an influenza pandemic	Nurse (*n* = 735) Hospital (*n* = NS) USA during H1N1 influenza pandemic	A total of 90.1% of nurses reported they would work during a pandemic. Willingness to work decreased with higher risk perception (PPE shortages, nurse’s workplace had to be quarantined and so on); family or nurse was perceived to be at risk and when vaccine or antiviral medication was not provided to both nurse and family. Ability to work decreased primarily when the nurse was sick, a loved one needed care at home or transportation problems existed.
18	Martin [43] (2013)	Quantitative descriptive	To examine potential predictors of nurses’ intentions to work during the 2009 influenza A (H1N1) pandemic	Nurse (*n* = 735) Hospital (*n* = NS ^1^) USA during H1N1 influenza pandemic	A total of 90% initially indicated that they intended to work during a flu pandemic. Nurses were more likely to work if provided with adequate PPE and less likely with inadequate PPE or if they feared family members could become ill with the pandemic flu. They were also less likely to work if assigned to direct care of a flu patient; if a colleague were quarantined or died of the pandemic flu; if they feared their own family members might die of pandemic flu; if they themselves were ill for any reason; if a family member or loved one were sick at home and needed care; if they lacked a written family protection plan; or if certain incentives were offered: antiviral medication or vaccine for nurse and family, double pay, or provided free room and board at work.
19	Nashwan [37] (2021)	Quantitative descriptive	To assess the role of nurses ’ knowledge and attitude in relation to their willingness to work with patients diagnosed with COVID-19 in Qatar	Nurse (*n* = 377) Hospital (*n* = 1) Qatar during COVID-19 pandemic	A total of 88.1% of the participants expressed their willingness to work with COVID-19 patients. Nurses with a higher level of knowledge about COVID-19 and infection control were more willing to work with COVID-19 patients (OR ^2^ = 0.874, CI ^4^: 0.766–0.996). Nurses who categorized themselves as low risk professionally, meaning indirectly supporting the COVID-19 pandemic through contributions such as office work, are less willing to care for patients with COVID-19 (OR ^2^ = 8.322, CI ^4^: 3.001–23.076).
20	Oh [38](2017)	Quantitativedescriptive	To examine levels of stress and professionalism of nurses who provided nursing care during the MERS outbreak and to investigate the nurses’ intentions to respond to possible future infectious disease outbreaks	Nurse (*n* = 313) Hospital (*n* = 5) South Korea using hypothetical situation	Factors significantly associated with nurses’ intention to provide care to patients with newly emerging infectious diseases included: 5 to 10 years of clinical experience compared with 5 years (β = −0.15, *p* < 0.05), MERS-treating hospitals with authorized beds compared with screening hospitals (β = 0.16, *p* < 0.01), outbreak nursing experience (β = 0.24, *p* < 0.01), stress (β = −0.21, *p* < 0.01), and professionalism in nursing (β = 0.23 *p* < 0.001).
21	Rafi [25] (2021)	Quantitative descriptive	To determine the prevalence and associated factors of willingness to work during the COVID-19 pandemic among the registered physicians of Bangladesh	Physicians (*n* = 313) Hospital (*n* = NS ^1^) Bangladesh during COVID-19 pandemic	A total of 69.7% of the participating physicians reported that they were willing to work during an initial COVID-19 lockdown. The factors affecting physicians’ willingness to work were age of 21–30 years (aOR ^5^ = 2.01, *p* < 0.01) and 31–40 years (aOR ^5^ = 2.11, *p* < 0.05), being a senior physician (consultant level to above) (aOR ^5^ = 0.01, *p* < 0.01), having experience of treating patients during previous pandemic (aOR ^5^ = 8.11, 95% CI ^4^: 1.80–36.52; *p* < 0.01), having experience of treating confirmed or suspected COVID-19 patients (aOR ^5^ = 0.11, *p* < 0.01), confidence in understanding how to protect themselves and their patients (aOR ^5^ = 2.43, *p* < 0.05), belief that using PPE would keep healthcare workers safe from getting COVID-19 (aOR ^5^ = 3.13, *p* < 0.05), high self-reported compliance to the recommended PPE (aOR ^5^ = 6.75, *p* < 0.05), and low self-perceived risk of being infected by SARS-CoV-2 from the workplace (aOR ^5^ = 2.85, *p* < 0.05). Working in the emergency departments, outpatient clinics, surgery/gynecology inpatient was positively related to willingness to work.
22	Sadang [36] (2021)	Qualitative	To explore and describe the meaning of nurses ’ work in the community quarantine facilities of Lanao del Sur Province amidst the COVID-19 pandemic	Nurse (*n* = 12) Hospital (*n* = NS ^1^) Philippines during COVID-19 pandemic	Themes identified: 1. Work as self-sacrifice: - Lack of personal protective equipment - Dealing with hundreds of clients daily - Working beyond the required hours 2. Work as self-fulfillment: - Opportunity to work and serve - Calling of their duty and profession 3. Work as psychological struggle - Presence of stigma as a health worker - Challenges in dealing with patients
23	Shaw [24] (2006)	Qualitative	To assess general practice preparedness to respond to an influenza pandemic and to identify issues that need to be addressed to enhance preparedness for the next pandemic	General practitioner (*n* = 60) Hospital (*n* = NS ^1^) Australia using hypothetical situation	Themes identified: 1. The role of the general practitioner in responding to pandemic influenza: general practitioners were primarily influenced by their sense of personal responsibility for their patients’ welfare and to their colleagues. Indemnity was specifically an issue for retired general practitioners and general practitioners not currently in clinical practice. 2. Practice preparedness issues: participants believed the government had a duty to provide PPE in the event of a pandemic. There was a lack of knowledge regarding prophylactic antivirals effective against the infectious agent. 3. The interface between general practice and the broader health sector: there was a need for competent leadership. 4. The expectations and requirements of general practitioners for the provision of professional services during a pandemic: general practitioners were enthusiastic about receiving further information and training in pandemic preparedness.
24	Shi [7] (2020)	Quantitative descriptive	To assess the knowledge and attitudes of medical staff in two Chinese mental health centers during the COVID-19 pandemic	Nurse (*n* = 170) (psychiatric nurse) Psychiatrist (*n* = 141) Hospital (*n* = 2) China during COVID-19 pandemic	Finishing a COVID-19 training program (OR ^2^ = 3.387, *p* < 0.001), experience of caring for patients with COVID-19 (OR ^2^ = 0.349, *p* = 0.018), confidence in knowing the risks (OR ^2^ = 2.978, *p* < 0.001), and knowing how to protect both themselves and patients (OR ^2^ = 2.889, *p* < 0.001) were associated with a likelihood of accepting a care assignment that included infected patients.
25	Simsek [42] (2021)	Qualitative	To examine the experiences and feelings of nurses who have children and are asked to care for patients with COVID-19	Nurse (*n* = 26) Hospital (*n* = 2) Turkey during COVID-19 pandemic	Themes identified: Longing: longing for children and longing for the pre-pandemic period Fear: fear of transmitting the disease and fear of death Despair Concern: concern resulting from working in a different clinic, concern resulting from lack of knowledge, and concern resulting from lack of protective equipment Professional responsibility: professional awareness and love for the profession
26	Tzeng [4] (2003)	Quantitative descriptive	To investigate the relation of hospital nurses’ willingness to provide care for severe acute respiratory syndrome (SARS) patients, their attitudes toward SARS infection control measures, nurses’ health status, and their demographic characteristics	Nurse (*n* = 126) Hospital (*n* = 6) Taiwan during SARS outbreak	Nurses’ positive attitudes toward infection control measures such as agreement with general SARS infection control measures (*p* = 0.016) and self-treatment of relief of fever and cough (*p* = 0.018) had a positive relationship with willingness to provide care for SARS patients. Necessity of closing hospitals (*p* = 0.037) had a negative relationship with nurses’ willingness to work.
27	Tzeng [41] (2004)	Quantitative descriptive	To characterize the changes in nurses’ perceptions of their professional care obligation and the relationship between hospital nurses’ professional obligation, their attitude toward SARS infection control measures, whether they had ever cared for patients with SARS, their current health status, select demographic characteristics, and the time of the data collection (during or after SARS)	Nurse (*n* = 112) Hospital (*n* = 6) Taiwan during SARS outbreak Nurse (*n* = 60) Hospital (*n* = 1) Taiwan after SARS outbreak	During a SARS outbreak, nurses’ level of agreement with general infection control measures was positively associated with nurses’ willingness to work. After a SARS outbreak, chronologically older nurses with fewer years of professional experience and nurses’ level of agreement with general infection control measures were both positively associated with nurses’ willingness to work. The need for quarantine after providing care for patients with SARS was negatively associated with nurses’ willingness to work. Overall, nurses’ levels of agreement with general SARS infection control measures had a positive relationship with nurses’ willingness, while the need for quarantine after providing care for infected patients had a negative relationship. After a SARS outbreak professional care obligations (during = 3.60, after = 3.91; *t* = −2.032, *p* = 0.044) and attitudes toward general SARS infection control measures (during = 3.99, after = 4.24; *t* = −3.114, *p* = 0.002) increased.
28	Tzeng [40] (2006)	Quantitative descriptive	To illustrate the factors that contribute to nurses’ fear about a possible AI pandemic and their willingness to care for patients infected with AI	Nurse (*n* = 225) Hospital (*n* = NS ^1^) Taiwan in high possibility of AI outbreak	Individuals’ religious activity (e.g., when you or a family member is ill, you would go to a temple or church to pray for help) and having sufficient infection control measures and equipment were positively associated with willingness to care for patients with AI (*p* = 0.017). Fear of a pandemic (e.g., you personally feel fearful about the bird flu epidemic) was negatively associated with willingness (*p* < 0.001).
29	Wong [33](2010)	Quantitativedescriptive	To explore the willingness of community-based nurses to continue to work during H1N1 influenza pandemic	Nurse (*n* = 270) Community setting Hong Kong during H1N1 influenza pandemic	Fear of infection (frightened of dealing with H1N1 influenza, worried about job-related infection), concern from family (your family is worried about being infected by you due to your job), family safety (worried about infecting your family due to your job,), and higher level of stress (e.g., influenza A (H1N1) affected your daily living activities, the quality of your life, feeling depressed and/or stressed) negatively affected willingness to work during H1N1 influenza pandemic (*p* < 0.001).

^1^ NS = not stated; ^2^ OR = odds ratio; ^3^ RR = relative risk; ^4^ CI = confidence interval; ^5^ aOR = adjusted odds ratio.

**Table 2:** Reciprocal translation table.

**Article Contents**	**Analytic Themes**				
	**Attitude**		**Perceived Norm**	**Environmental Constraints**	**Habit**
Anikeeva [23] (2008)	Antiviral medication: families having access to the medication is important to feel prepared to work in an influenza pandemic. General practitioners’ justification of planned actions: general sense of commitment to the public good, moral obligation vs. responsibility to oneself to stay healthy and to protect their own family.		General practitioners’ justification of planned actions: general sense of commitment to the public good, moral obligation vs. responsibility to oneself to stay healthy and to protect one’s own family.	Personal protective equipment (PPE): the government has a reciprocal duty to ensure that working conditions are safe	
Cui [27] (2020)			Motivations for supporting the hardest-hit areas: professional commitment, media propaganda		
Kim [39] (2018)	Hesitancy and hoping to avoid: Nurses did not want to work due to fear of infection, but they were compelled to go contribute. Remaining task -Futility of forgotten warriors: nurses felt that compensation and rewards were not adequate given the work expectations and their professional efforts.		Strong responsibility as a nurse to care for patients during a pandemic.		Growth as a nurse -Constant mind control, meaning the nurses focused their thoughts on being safe because they had PPE and felt mentally strong due to working in an extreme situation.
Article Contents	Analytic Themes				
	Attitude	Perceived norm	Environmental constraints	Habit	
Liu [2] (2020)		A call to duty to care for patients during a pandemic.			
Liu [30] (2020)		Strong responsibility and identity as a healthcare provider: -Responsibility and mission as a healthcare provider			
Sadang [36] (2021)		Work as self-fulfillment: -Opportunity to work and serve -Calling of their duty and profession			
Shaw [24] (2006)	The role of the general practitioner in responding to pandemic: Indemnity (meaning, protection from legal liability; specifically as an issue for retired general practitioners and general practitioners not currently in clinical practice).	The role of the general practitioner in responding to a pandemic: general practitioners were primarily influenced by their sense of personal responsibility for their patients’ welfare as well as their colleagues.	Practice preparedness issues: the government had a duty to provide adequate PPE in the event of a pandemic		
Simsek [42] (2021)		Professional responsibility: professional awareness and love for the profession			

## Data Availability

Not applicable.

## Supplementary Materials

The following are available online at https://www.mdpi.com/article/10.3390/ijerph18136841/s1, Table S1: Literature search strategy, Table S2: Mixed Method Appraisal Tool (MMAT) in selected studies (N = 29).
