# Supplementary material for: Barriers and Facilitators of Nurses’ and Physicians’ Willingness to Work during a Respiratory Disease Outbreak: A Mixed-Methods Systematic Review
Source: Int J Environ Res Public Health. 2021 Jun 25;18(13):6841. doi: 10.3390/ijerph18136841 (PMC8296986; doi:10.3390/ijerph18136841)
Supplement: Supplementary file 1 [file ijerph-18-06841-s001.zip › ijerph-1263709-supplementary.pdf]

Table S1. Literature search strategy

Database	Search terms	N
PubMed (until 31th March 2021)	((nurse OR nurses OR physician OR physicians OR "healthcare worker" OR "healthcare workers" OR "healthcare provider" OR "healthcare providers" OR "health personnel" OR "nurses"[MeSH Terms] OR "physicians"[MeSH Terms] OR "health personnel"[MeSH Terms]) AND (Pandemic OR Pandemics OR "Healthcare disaster" OR (respiratory AND "infectious disease" AND epidemic) OR "Disease Outbreak*" OR "pandemics"[MeSH Terms])) AND (Willingness OR (Willing* AND work) OR "Report to work" OR (Respon* AND work))	978
Web of Science (until 5th April 2021)	All field: (nurse OR nurses OR physician OR physicians OR "healthcare worker" OR "healthcare workers" OR "healthcare provider" OR "healthcare providers" OR "health personnel") AND All field: (Pandemic OR Pandemics OR "Healthcare disaster" OR (respiratory AND "infectious disease" AND epidemic) OR "Disease Outbreak*") AND All field: (Willingness OR (Willing* AND work) OR "Report to work" OR (Respon* AND work))	635
CINAHL (until 31th March 2021)	(nurse OR nurses OR physician OR physicians OR "healthcare worker" OR "healthcare workers" OR "healthcare provider" OR "healthcare providers" OR "health personnel" OR "nurses"[Word in Subject Heading] OR "physicians"[Word in Subject Heading]) AND (Pandemic OR Pandemics OR "Healthcare disaster" OR (respiratory AND "infectious disease" AND epidemic) OR "Disease Outbreaks"[Word in Subject Heading]) AND (Willingness OR (Willing* AND work) OR "Report to work" OR (Respon* AND work))	311
PsycINFO (until 31th March 2021)	(nurse OR nurses OR physician OR physicians OR "healthcare worker" OR "healthcare workers" OR "healthcare provider" OR "healthcare providers" OR "health personnel" OR MAINSUBJECT.EXACT("nurses") OR MAINSUBJECT.EXACT("physicians") OR MAINSUBJECT.EXACT ("health personnel")) AND (Pandemic OR Pandemics OR "Healthcare disaster" OR (respiratory AND "infectious disease" AND epidemic) OR ("disease outbreak" OR "disease outbreaks") OR MAINSUBJECT.EXACT("pandemics")) AND (Willingness OR (Willing* AND work) OR "Report to work" OR (Respon* AND work))	97

Table S2. Mixed Method Appraisal Tool (MMAT) in selected studies (N=29)

First Author (Year)	Design	Methodological quality criteria	Yes	No	Cannot tell	Comment
**Adam (2014)**	Screening questions (for all types)	1. Are there clear research questions?	✓			No information about measurements
		2. Do the collected data allow to address the research questions?	✓			
	Quantitative descriptive	4.1. Is the sampling strategy relevant to address the research question?	✓			
		4.2. Is the sample representative of the target population?	✓			
		4.3. Are the measurements appropriate?			✓	
		4.4. Is the risk of nonresponse bias low?	✓			
		4.5. Is the statistical analysis appropriate to answer the research question?	✓			
**Al-Hunaish (2019)**	Screening questions (for all types)	1. Are there clear research questions?	✓			
		2. Do the collected data allow to address the research questions?	✓			
	Quantitative descriptive	4.1. Is the sampling strategy relevant to address the research question?	✓			
		4.2. Is the sample representative of the target population?	✓			
		4.3. Are the measurements appropriate?	✓			
		4.4. Is the risk of nonresponse bias low?	✓			
		4.5. Is the statistical analysis appropriate to answer the research question?	✓			
**Anikeeva (2008)**	Screening questions (for all types)	1. Are there clear research questions?	✓			
		2. Do the collected data allow to address the research questions?	✓			
	Qualitative	1.1. Is the qualitative approach appropriate to answer the research question?	✓			
		1.2. Are the qualitative data collection methods adequate to address the research question?	✓			

		1.3. Are the findings adequately derived from the data?	✓			
		1.4. Is the interpretation of results sufficiently substantiated by data?	✓			
		1.5. Is there coherence between qualitative data sources, collection, analysis, and interpretation?	✓			
**Ayub (2020)**	Screening questions (for all types)	1. Are there clear research questions?	✓			No information about reliability and validity test of measurements
		2. Do the collected data allow to address the research questions?	✓			
	Quantitative descriptive	4.1. Is the sampling strategy relevant to address the research question?	✓			
		4.2. Is the sample representative of the target population?	✓			
		4.3. Are the measurements appropriate?			✓	
		4.4. Is the risk of nonresponse bias low?	✓			
		4.5. Is the statistical analysis appropriate to answer the research question?	✓			
**Bell (2014)**	Screening questions (for all types)	1. Are there clear research questions?	✓			Response rate was low, 46%
		2. Do the collected data allow to address the research questions?	✓			
	Quantitative descriptive	4.1. Is the sampling strategy relevant to address the research question?	✓			
		4.2. Is the sample representative of the target population?	✓			
		4.3. Are the measurements appropriate?	✓			
		4.4. Is the risk of nonresponse bias low?		✓		
		4.5. Is the statistical analysis appropriate to answer the research question?	✓			
**Butsashvili (2007)**	Screening questions (for all types)	1. Are there clear research questions?	✓			No information about sampling procedure, reliability test results of
		2. Do the collected data allow to address the research questions?	✓			
	Quantitative descriptive	4.1. Is the sampling strategy relevant to address the research question?	✓			
		4.2. Is the sample representative of the target population?			✓	

		4.3. Are the measurements appropriate?			✓	measurement and nonresponse
		4.4. Is the risk of nonresponse bias low?			✓	
		4.5. Is the statistical analysis appropriate to answer the research question?	✓			
**Cui (2020)**	Screening questions (for all types)	1. Are there clear research questions?	✓			
		2. Do the collected data allow to address the research questions?	✓			
	Qualitative	1.1. Is the qualitative approach appropriate to answer the research question?	✓			
		1.2. Are the qualitative data collection methods adequate to address the research question?	✓			
		1.3. Are the findings adequately derived from the data?	✓			
		1.4. Is the interpretation of results sufficiently substantiated by data?	✓			
		1.5. Is there coherence between qualitative data sources, collection, analysis, and interpretation?	✓			
**Dickinson (2013)**	Screening questions (for all types)	1. Are there clear research questions?	✓			No information about reliability and validity test of measurements<br><br>Response rate was low, 22%
		2. Do the collected data allow to address the research questions?	✓			
	Quantitative descriptive	4.1. Is the sampling strategy relevant to address the research question?	✓			
		4.2. Is the sample representative of the target population?	✓			
		4.3. Are the measurements appropriate?			✓	
		4.4. Is the risk of nonresponse bias low?		✓		
		4.5. Is the statistical analysis appropriate to answer the research question?	✓			
**Gan (2020)**	Screening questions (for all types)	1. Are there clear research questions?	✓			
		2. Do the collected data allow to address the research questions?	✓			
	Quantitative descriptive	4.1. Is the sampling strategy relevant to address the research question?	✓			
		4.2. Is the sample representative of the target population?	✓			

		4.3. Are the measurements appropriate?	✓			
		4.4. Is the risk of nonresponse bias low?	✓			
		4.5. Is the statistical analysis appropriate to answer the research question?	✓			
**Hope (2011)**	Screening questions (for all types)	1. Are there clear research questions?	✓			No information about inclusion and exclusion criteria of sample<br><br>Lack of information about measurements<br><br>No information about confounders
		2. Do the collected data allow to address the research questions?	✓			
	Quantitative non-randomized	3.1. Are the participants representative of the target population?			✓	
		3.2. Are measurements appropriate regarding both the outcome and intervention (or exposure)?			✓	
		3.3. Are there complete outcome data?	✓			
		3.4. Are the confounders accounted for in the design and analysis?		✓		
		3.5 During the study period, is the intervention administered (or exposure occurred) as intended?	✓			
**Kim (2018)**	Screening questions (for all types)	1. Are there clear research questions?	✓			
		2. Do the collected data allow to address the research questions?	✓			
	Qualitative	1.1. Is the qualitative approach appropriate to answer the research question?	✓			
		1.2. Are the qualitative data collection methods adequate to address the research question?	✓			
		1.3. Are the findings adequately derived from the data?	✓			
		1.4. Is the interpretation of results sufficiently substantiated by data?	✓			
		1.5. Is there coherence between qualitative data sources, collection, analysis, and interpretation?	✓			
**Li (2020)**	Screening questions (for all types)	1. Are there clear research questions?	✓			Lack of information about sampling strategy
		2. Do the collected data allow to address the research questions?	✓			
		4.1. Is the sampling strategy relevant to address the research question?			✓	

	Quantitative descriptive	4.2. Is the sample representative of the target population?	✓			
		4.3. Are the measurements appropriate?	✓			
		4.4. Is the risk of nonresponse bias low?	✓			
		4.5. Is the statistical analysis appropriate to answer the research question?	✓			
**Liu (2020)** [a]	Screening questions (for all types)	1. Are there clear research questions?	✓			
		2. Do the collected data allow to address the research questions?	✓			
	Qualitative	1.1. Is the qualitative approach appropriate to answer the research question?	✓			
		1.2. Are the qualitative data collection methods adequate to address the research question?	✓			
		1.3. Are the findings adequately derived from the data?	✓			
		1.4. Is the interpretation of results sufficiently substantiated by data?	✓			
		1.5. Is there coherence between qualitative data sources, collection, analysis, and interpretation?	✓			
**Liu (2020)** [b]	Screening questions (for all types)	1. Are there clear research questions?	✓			
		2. Do the collected data allow to address the research questions?	✓			
	Qualitative	1.1. Is the qualitative approach appropriate to answer the research question?	✓			
		1.2. Are the qualitative data collection methods adequate to address the research question?	✓			
		1.3. Are the findings adequately derived from the data?	✓			
		1.4. Is the interpretation of results sufficiently substantiated by data?	✓			
		1.5. Is there coherence between qualitative data sources, collection, analysis, and interpretation?	✓			
**Lord (2021)**[a]	Screening questions (for all types)	1. Are there clear research questions?	✓			Small number of sample (n=83)
		2. Do the collected data allow to address the research questions?	✓			

	Quantitative descriptive	4.1. Is the sampling strategy relevant to address the research question?	✓			Lack of information about measurements<br><br>Response rate was low, 42%
		4.2. Is the sample representative of the target population?			✓	
		4.3. Are the measurements appropriate?			✓	
		4.4. Is the risk of nonresponse bias low?		✓		
		4.5. Is the statistical analysis appropriate to answer the research question?	✓			
**Luo (2021)**	Screening questions (for all types)	1. Are there clear research questions?	✓			
		2. Do the collected data allow to address the research questions?	✓			
	Quantitative descriptive	4.1. Is the sampling strategy relevant to address the research question?	✓			
		4.2. Is the sample representative of the target population?	✓			
		4.3. Are the measurements appropriate?	✓			
		4.4. Is the risk of nonresponse bias low?	✓			
		4.5. Is the statistical analysis appropriate to answer the research question?	✓			
**Martin (2011)**	Screening questions (for all types)	1. Are there clear research questions?	✓			
		2. Do the collected data allow to address the research questions?	✓			
	Quantitative descriptive	4.1. Is the sampling strategy relevant to address the research question?	✓			
		4.2. Is the sample representative of the target population?	✓			
		4.3. Are the measurements appropriate?	✓			
		4.4. Is the risk of nonresponse bias low?	✓			
		4.5. Is the statistical analysis appropriate to answer the research question?	✓			
**Martin (2013)**	Screening questions (for all types)	1. Are there clear research questions?	✓			
		2. Do the collected data allow to address the research questions?	✓			
		4.1. Is the sampling strategy relevant to address the research question?	✓			

	Quantitative descriptive	4.2. Is the sample representative of the target population?	✓			
		4.3. Are the measurements appropriate?	✓			
		4.4. Is the risk of nonresponse bias low?	✓			
		4.5. Is the statistical analysis appropriate to answer the research question?	✓			
**Nashwan (2021)**	Screening questions (for all types)	1. Are there clear research questions?	✓			No information about inclusion and exclusion criteria of sample<br><br>No information about translation process of measurement
		2. Do the collected data allow to address the research questions?	✓			
	Quantitative descriptive	4.1. Is the sampling strategy relevant to address the research question?	✓			
		4.2. Is the sample representative of the target population?			✓	
		4.3. Are the measurements appropriate?			✓	
		4.4. Is the risk of nonresponse bias low?	✓			
		4.5. Is the statistical analysis appropriate to answer the research question?	✓			
**Oh (2017)**	Screening questions (for all types)	1. Are there clear research questions?	✓			
		2. Do the collected data allow to address the research questions?	✓			
	Quantitative descriptive	4.1. Is the sampling strategy relevant to address the research question?	✓			
		4.2. Is the sample representative of the target population?	✓			
		4.3. Are the measurements appropriate?	✓			
		4.4. Is the risk of nonresponse bias low?	✓			
		4.5. Is the statistical analysis appropriate to answer the research question?	✓			
**Rafi (2021)**	Screening questions (for all types)	1. Are there clear research questions?	✓			No information about reliability and validity test of measurements
		2. Do the collected data allow to address the research questions?	✓			
	Quantitative descriptive	4.1. Is the sampling strategy relevant to address the research question?	✓			
		4.2. Is the sample representative of the target population?	✓			

		4.3. Are the measurements appropriate?			✓	
		4.4. Is the risk of nonresponse bias low?	✓			
		4.5. Is the statistical analysis appropriate to answer the research question?	✓			
**Sadang (2020)**	Screening questions (for all types)	1. Are there clear research questions?	✓			
		2. Do the collected data allow to address the research questions?	✓			
	Qualitative	1.1. Is the qualitative approach appropriate to answer the research question?	✓			
		1.2. Are the qualitative data collection methods adequate to address the research question?	✓			
		1.3. Are the findings adequately derived from the data?	✓			
		1.4. Is the interpretation of results sufficiently substantiated by data?	✓			
		1.5. Is there coherence between qualitative data sources, collection, analysis, and interpretation?	✓			
**Shaw (2006)**	Screening questions (for all types)	1. Are there clear research questions?	✓			Lack of information about analysis
		2. Do the collected data allow to address the research questions?	✓			
	Qualitative	1.1. Is the qualitative approach appropriate to answer the research question?	✓			
		1.2. Are the qualitative data collection methods adequate to address the research question?	✓			
		1.3. Are the findings adequately derived from the data?			✓	
		1.4. Is the interpretation of results sufficiently substantiated by data?	✓			
		1.5. Is there coherence between qualitative data sources, collection, analysis, and interpretation?	✓			
**Shi (2020)**	Screening questions (for all types)	1. Are there clear research questions?	✓			No information about inclusion and exclusion criteria of sample
		2. Do the collected data allow to address the research questions?	✓			
		4.1. Is the sampling strategy relevant to address the research question?	✓			

	Quantitative descriptive	4.2. Is the sample representative of the target population?			✓	No information about reliability and validity test of measurements
		4.3. Are the measurements appropriate?			✓	
		4.4. Is the risk of nonresponse bias low?	✓			
		4.5. Is the statistical analysis appropriate to answer the research question?	✓			
**Simsek (2021)**	Screening questions (for all types)	1. Are there clear research questions?	✓			Only using documentation not participant interviews<br><br>Inconsistency between identified theme and supporting quotations
		2. Do the collected data allow to address the research questions?	✓			
	Qualitative	1.1. Is the qualitative approach appropriate to answer the research question?	✓			
		1.2. Are the qualitative data collection methods adequate to address the research question?			✓	
		1.3. Are the findings adequately derived from the data?		✓		
		1.4. Is the interpretation of results sufficiently substantiated by data?	✓			
		1.5. Is there coherence between qualitative data sources, collection, analysis, and interpretation?	✓			
**Tzeng (2003)**	Screening questions (for all types)	1. Are there clear research questions?	✓			No information about response rate
		2. Do the collected data allow to address the research questions?	✓			
	Quantitative descriptive	4.1. Is the sampling strategy relevant to address the research question?	✓			
		4.2. Is the sample representative of the target population?	✓			
		4.3. Are the measurements appropriate?	✓			
		4.4. Is the risk of nonresponse bias low?			✓	
		4.5. Is the statistical analysis appropriate to answer the research question?	✓			
**Tzeng (2004)**	Screening questions (for all types)	1. Are there clear research questions?	✓			No information about response rate
		2. Do the collected data allow to address the research questions?	✓			

	Quantitative descriptive	4.1. Is the sampling strategy relevant to address the research question?	✓			
		4.2. Is the sample representative of the target population?	✓			
		4.3. Are the measurements appropriate?	✓			
		4.4. Is the risk of nonresponse bias low?			✓	
		4.5. Is the statistical analysis appropriate to answer the research question?	✓			
**Tzeng (2006)**	Screening questions (for all types)	1. Are there clear research questions?	✓			
		2. Do the collected data allow to address the research questions?	✓			
	Quantitative descriptive	4.1. Is the sampling strategy relevant to address the research question?	✓			
		4.2. Is the sample representative of the target population?	✓			
		4.3. Are the measurements appropriate?	✓			
		4.4. Is the risk of nonresponse bias low?	✓			
		4.5. Is the statistical analysis appropriate to answer the research question?	✓			
**Wong (2010)**	Screening questions (for all types)	1. Are there clear research questions?	✓			Lack of information about measurements
		2. Do the collected data allow to address the research questions?	✓			
	Quantitative descriptive	4.1. Is the sampling strategy relevant to address the research question?	✓			
		4.2. Is the sample representative of the target population?	✓			
		4.3. Are the measurements appropriate?			✓	
		4.4. Is the risk of nonresponse bias low?	✓			
		4.5. Is the statistical analysis appropriate to answer the research question?	✓			
